# Microchip capillary gel electrophoresis combined with lectin affinity enrichment employing magnetic beads for glycoprotein analysis

**DOI:** 10.1007/s00216-017-0615-0

**Published:** 2017-09-20

**Authors:** Nicole Y. Engel, Victor U. Weiss, Christian Wenz, Susanne Glück, Andreas Rüfer, Martin Kratzmeier, Martina Marchetti-Deschmann, Günter Allmaier

**Affiliations:** 1 Institute of Chemical Technologies and Analytics, Vienna University of Technology, Getreidemarkt 9/164-IAC, 1060 Vienna, Austria; 2 Agilent Technologies, Hewlett-Packard-Straße 8, 76337 Waldbronn, Germany

**Keywords:** Affinity enrichment, Glycoprotein, Lab-on-a-chip, Lectin, Magnetic beads

## Abstract

Due to the constant search for reliable methods to investigate glycoproteins in complex biological samples, an alternative approach combining affinity enrichment with rapid and sensitive analysis on-a-chip is presented. Glycoproteins were specifically captured by lectin-coated magnetic beads, eluted by competitive sugars, and investigated with microchip capillary gel electrophoresis (MCGE), i.e., CGE-on-a-chip. We compared our results to sodium dodecyl sulfate-polyacrylamide gel electrophoresis (SDS-PAGE) data, which turned out to be in very good agreement. While SDS-PAGE offers the possibility of subsequent mass spectrometric analysis of captured and separated analytes, MCGE scores with time savings, higher throughput, and lower sample consumption as well as quality control (QC) and process analytical technology (PAT) applicability. Due to these advantages, a lectin-based glycoprotein capture protocol can easily be optimized. In our case, two different types of magnetic beads were tested and compared regarding lectin binding. The selectivity of our strategy was demonstrated with a set of model glycoproteins, as well as with human serum and serum depleted from high-abundance proteins. The specificity of the capturing method was investigated revealing to a certain degree an unspecific binding between each sample and the beads themselves, which has to be considered for any specific enrichment and data interpretation. In addition, two glycoproteins from *Trichoderma atroviride*, a fungus with mycoparasitic activity and only barely studied glycoproteome, were enriched by means of a lectin and so identified for the first time.

**Electronic supplementary material:**

The online version of this article (10.1007/s00216-017-0615-0) contains supplementary material, which is available to authorized users.

**Graphical abstract:**
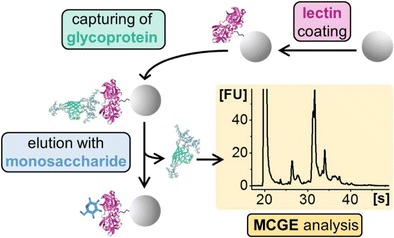
Glycoproteins from biological samples were detected by microchip capillary gel electrophoresis after lectin affinity enrichment using magnetic beads and elution with respective competitive monosaccharides

## Introduction

Glycosylations are regarded the most complex and, on the other hand, most common type of posttranslational modifications with more than 50% of all eukaryotic proteins being glycoproteins [1]. Glycoprotein analysis can be difficult due to their considerable macro- and microheterogeneity: Attached sugar moieties can range from single monosaccharides to complex linear or branched oligo- or even polysaccharides. Their huge structural diversity is also reflected in the variety of their functional purposes, which include their role in many molecular recognition events [2].

Carbohydrate-binding proteins like lectins play nowadays an important role in the structural and functional elucidation of glycoproteins, as well as in the study of their binding affinities and interactions with other proteins [3, 4]. In contrast to antibodies, lectins are generally more stable, more affordable, better characterized, and address a broader spectrum of glycoproteins. On the other hand, they have lower binding specificities, which, however, can be of advantage for certain analytical strategies and application areas. One of their main fields of application is lectin affinity chromatography, during which they can be used for the specific and selective isolation of certain glycoproteins of interest [5, 6]. Therefore, lectins are immobilized on, e.g., agarose- or silica-based media and filled into chromatographic columns of quite different dimensions. Typical lectins in this context are Concanavalin A (ConA), wheat germ agglutinin (WGA), and *Sambucus nigra* agglutinin (SNA). ConA specifically recognizes the trimannosidic core structure of an *N*-glycoprotein and other high-mannose structures [7], WGA binds to terminal *N*-acetylglucosamine and its β(1,4)-linked oligomers [8], and SNA has a high affinity for sialic acids (*N*-acetylneuraminic acid) α-glycosidically linked to galactose or *N*-acetylgalactosamine [9].

Today also other solid supports like magnetic beads are used especially for glycoprotein enrichment from crude biological samples without a chromatographic setup [10]. They allow working directly from small sample amounts without elaborate sample preparation and simplify sample washing as well as enrichment. In order to enrich a great variety of glycoproteins, lectins having a broader specificity can be combined [11]. Therefore, a combination of, e.g., ConA, WGA, and SNA enables the enrichment of a wide range of different *N*-glycoproteins based on the lectins’ specificities for common structural *N*-glycan moieties.

For investigation of isolated glycoproteins, the specific lectin affinity enrichment step has to be combined with an appropriate analytical technique. Very often, gel electrophoresis, especially sodium dodecyl sulfate- polyacrylamide gel electrophoresis (SDS-PAGE), is chosen for a first insight into enrichment efficiency. Although being a generally established method and allowing subsequent analyte identification via mass spectrometry after separation, SDS-PAGE has some major drawbacks. Next to being very time-consuming, it lacks automation and therefore high throughput and requires certain technical skills, i.e., manual handling and the uniform staining of glycoproteins after separation can be influenced by their modifications hampering quantification. Microchip capillary gel electrophoresis (MCGE), on the other hand, offers rapid (1 min per sample) and reliable size-based separations in addition to be performed in a high throughput fashion and quantitation in real time [12–14]. As a microfluidic system, it combines sample handling, separation of analytes, staining, laser-induced fluorescence (LIF) detection, and data analysis on one chip. Depending on the type of fluorescence labeling, available protein assays feature sensitivities comparable to silver-stained (covalent pre-chip labeling) or Coomassie-stained (dynamic on-chip labeling) gels. Both available MCGE protein assays have already been characterized in regard to glycoprotein sizing, limits of detection, and quantitation [15].

In this work for the first time, glycoprotein investigation by MCGE was successfully combined with a preceding specific lectin affinity enrichment using magnetic beads. Due to the speed of MCGE analysis, its low sample consumption, and the ease of quantification of obtained results, it was possible to study lectin affinity enrichment protocols in detail, which is a necessary prerequisite for subsequent glycoprotein enrichment from complex samples. Thus, our approach significantly contributes to method development. For optimization of the presented approach in our case, two magnetic beads from different providers based on different basic chemical composition (silica and polymer) were tested and compared, namely MagSi-S Tosyl 1.0 (MagSi) beads and tosylactivated Dynabeads MyOne (Dynabeads). Based on preliminary investigations, detailed binding and elution conditions were evaluated finally for Dynabeads with a set of model lectins and glycoproteins of varying molecular weight and degree of glycosylation. For validation of the method and investigation of its selectivity, complex biological samples (human blood serum and mycelia derived from *Trichoderma atroviride*) were used. All results were compared to traditional SDS-PAGE analysis. Additionally, enriched glycoproteins were identified by tryptic *in-gel* digestion and subsequent matrix-assisted laser desorption/ionization time-of-flight mass spectrometry (MALDI-TOF-MS).

## Materials and methods

### Materials

Human serum transferrin (TF, ≥ 98%), bovine acid glycoprotein (AGP, 99%), human antitrypsin (A1AT, salt free, lyophilized powder), β-galactosidase from *E. coli* (β-Gal, lyophilized powder), bovine serum albumin (BSA, ≥ 96%), as well as all other chemicals (purity of at least 99%, each) were purchased from Sigma-Aldrich (St. Louis, MO, USA), if not stated otherwise. Sodium thiosulfate pentahydrate, ammonium sulfate, ethanol, acetonitrile (ACN), and acetic acid (all analytical grade) were obtained from Merck (Darmstadt, Germany). NuPAGE 4–12% Bis-Tris gels, 4× lithium dodecyl sulfate (LDS) sample buffer (106 mM Tris HCl, 141 mM Tris Base, 2% LDS, 10% glycerol, 0.51 mM EDTA, 0.22 mM Serva Blue G250, 0.175 mM Phenol Red, pH 8.5), 20× MES SDS running buffer (50 mM MES, 50 mM Tris Base, 0.1% SDS, 1 mM EDTA, pH 7.3), and BenchMark Protein Ladder were acquired from Life Technologies (Carlsbad, CA, USA). ConA, WGA, and SNA were obtained from Vector Laboratories (Burlingame, CA, USA). Dynabeads MyOne Tosylactivated were from Invitrogen Dynal (Oslo, Norway) and MagSi-S Tosyl 1.0 beads from MagnaMedics (Geleen, The Netherlands). The Agilent 2100 Bioanalyzer, the Protein 230 (P230), and the High Sensitivity Protein 250 (HSP-250) Kit were obtained from Agilent Technologies (Waldbronn, Germany). Sequencing grade trypsin from bovine was from Roche (Mannheim, Germany). All experiments were performed employing water of Millipore grade (18.2 MΩ cm resistivity at 25 °C) taken from a Simplicity system (Millipore, Molsheim, France).

### Buffers

Lectins were dissolved in 0.1 M sodium borate and 0.1 mM CaCl_2_ (pH 8.3) to 100 μM and stored at 4 °C until usage. Coupling buffer (0.1 M sodium borate, 1 M ammonium sulfate, pH 9.5) was freshly prepared. Blocking buffer (1 M Tris, 150 mM NaCl, 1 mM CaCl_2_, 1 mM MnCl_2_, pH 7.4) and binding buffer (20 mM Tris, 150 mM NaCl, 1 mM CaCl_2_, 1 mM MnCl_2_, pH 7.4) were prepared and stored at 4 °C for no longer than 2 weeks. pH was checked prior to application.

### Sample preparation

Human blood was taken from a healthy, voluntary donor with a sterile lancet and centrifuged at 14,000×*g*, for 30 min. The serum was stored at −20 °C. According to the manufacturer’s operating procedure, 10 μL of serum was incubated in a Pierce Top 12 Abundant Protein Depletion Spin Column (Thermo Fisher Scientific, Waltham, USA) for 1 h to deplete high-abundance proteins. The filtrate was concentrated and its buffer exchanged to binding buffer using 10 kDa Millipore Microcon centrifugal filters (Merck Millipore, Darmstadt, Germany) with an Ultracel regenerated cellulose membrane.

In the case of *T. atroviride*, frozen mycelia was equilibrated to ambient temperature before analysis. Of wet cell mycelia, 100 mg was suspended in 1.6 mL binding buffer and lysed by sonication (intensity 60%, 2× 20 s and 1× 30 s, 1 min waiting intervals each, at 4 °C) with a Branson Sonifier 250 (Branson Ultrasonics, Danbury, CT, USA). Lysed cells were centrifuged (14,000×*g*, 4 °C, 20 min), the supernatant was collected and concentrated by means of 3 kDa Millipore Microcon centrifugal filters.

Protein concentrations of all samples were determined using Bradford Assay and BSA for calibration.

### Glycoprotein enrichment

Magnetic beads were handled as suggested by the manufacturer. Briefly, 5.33 μM lectin was covalently linked to 20 μg/μL tosylactivated magnetic beads in coupling buffer at 37 °C and 600 rpm for 20 h. Free tosyl groups were inactivated by overnight incubation in blocking buffer at 37 °C and 600 rpm. The prepared beads were washed two times with a fivefold volume of binding buffer and stored in the same buffer at 4 °C. Prepared beads were found to be stable for at least 2 weeks and were used within this time period.

Glycoprotein samples (0.5 μg/μL standard proteins, 4.7 μg/μL serum, 2.0 μg/μL depleted serum, 4.7 μg/μL *T. atroviride*) were incubated with 20 μg/μL lectin beads in binding buffer at room temperature and 600 rpm for 60 min to specifically capture the glycoproteins. The beads with captured glycoproteins were washed three times with fivefold volume of binding buffer. Glycoproteins were eluted from the beads with the respective competitive mono- and disaccharide of the lectin at room temperature for 15 min and 600 rpm. Methyl α-d-glucopyranoside and methyl α-d-mannopyranoside, 200 mM each, were used for ConA, 500 mM *N*-acetyl-d-glucosamine for WGA, and 500 mM lactose for SNA. Elution of remaining non-covalently bound analytes was achieved by incubating the beads for 5 min at 95 °C either with 5 μL 4× LDS sample buffer containing 50 mM DTT for SDS-PAGE or with 6 μL P230/HSP-250 sample buffer (diluted according to manufacturer’s protocol in water) containing 11.7 mM DTT for MCGE.

### MCGE

The chip-based glycoprotein analysis was performed on the Agilent 2100 Bioanalyzer system either with the P230 or the HSP-250 assay according to the manufacturer’s instructions. Briefly, samples were fluorescently labeled for the HSP-250 assay prior to denaturation. Sample solution (10 μL) was mixed with 1 μL HSP-250 labeling dye and incubated on ice for 30 min. The labeling reaction was stopped by adding 1 μL of ethanolamine followed by incubation on ice for additional 10 min. No labeling was necessary for samples analyzed by the P230 assay. Samples were diluted only with water prior to denaturation. For this, 4 μL sample was incubated (95 °C, 5 min) with 2 μL sample buffer containing DTT. HSP-250 assay samples were directly applied to the chip, whereas P230 assay samples were diluted with 84 μL water before application.

### SDS-PAGE

SDS-PAGE analyses were carried out on NuPAGE 4–12% Bis-Tris gels using MES SDS running buffer at 120 V (const.) and 60 mA (max.). BenchMark Protein Ladder was applied for molecular weight determination. Protein bands were visualized by silver staining suited for further MS analyses [16]. Briefly, gels were washed with an aqueous solution containing 50% ethanol and 5% acetic acid for 20 min, with 50% ethanol for 10 min, and three times with water for 20 min each. Afterwards, gels were incubated in 0.2% sodium thiosulfate pentahydrate for 1 min and washed two times with water for 1 min. Subsequently, gels were incubated in 1% silver nitrate at 4 °C for 20 min and washed twice with water for 1 min. Gels were further incubated in 2% sodium carbonate containing 0.04% formaldehyde until protein bands were visible. Staining was stopped with 5% acetic acid. Gels were stored in 1% acetic acid at 4 °C for further investigations.

### Tryptic *in-gel* digestion and MALDI mass spectrometric identification

After SDS-PAGE glycoproteins were identified by MALDI Reflectron TOF MS (MALDI-RTOF-MS) and MALDI-MS/MS analyses after destaining the gel and tryptic *in-gel* digestion. Briefly, excised gel bands were destained in 100 mM Na_2_S_2_O_3_/30 mM K_4_Fe(CN)_6_·3H_2_O (1:1, *v*/*v*) [17]. Gel pieces were treated with ACN, rehydrated (100 mM NH_4_HCO_3_), reduced (10 mM DTT in 100 mM NH_4_HCO_3_, 56 °C, 45 min), alkylated (50 mM iodoacetamide in 100 mM NH_4_HCO_3_, 24 °C, 30 min), and finally dried in a vacuum centrifuge. After rehydration in approx. 10 μL 50 mM NH_4_HCO_3_ (pH 8.5) containing 5% ACN and 100 ng trypsin and incubation at 37 °C overnight, peptides were extracted with 50 mM NH_4_HCO_3_/ACN (1:1, *v*/*v*) and 0.1% formic acid/ACN (1:1, *v*/*v*). All extracts of selected lanes were dried in a vacuum centrifuge. After reconstitution in 0.1% aqueous TFA, peptides were desalted using C_18_ ZipTip® pipette tips (Merck Millipore) and eluted with 2.7 μg/μL α-cyano-4-hydroxycinnamic acid (MALDI-MS matrix) prepared in aqueous ACN (50%) containing 0.1% TFA. Peptide mass fingerprinting (PMF) and MS/MS analyses were carried out on an UltrafleXtreme MALDI-linear TOF/RTOF instrument using an AnchorChip target (both Bruker Daltonik, Bremen, Germany).

For all data related to enzymatic digestion, autolytic tryptic products, keratin, and blank artifacts were assigned and removed before database search (SWISS-PROT with taxonomy *Homo sapiens* for human samples and in all entries of NCBInr for *Trichoderma* samples, September–December 2013) using Mascot [18] with the following parameters: monoisotopic mass values, peptide mass tolerance of ± 0.15 and 0.3 Da for PMF and MS/MS experiments, respectively, one missed cleavage, carbamidomethylation as fixed modification, and methionine oxidations and methylation of the protein N-terminus as variable modifications.

## Results and discussion

**Fig. 1:**
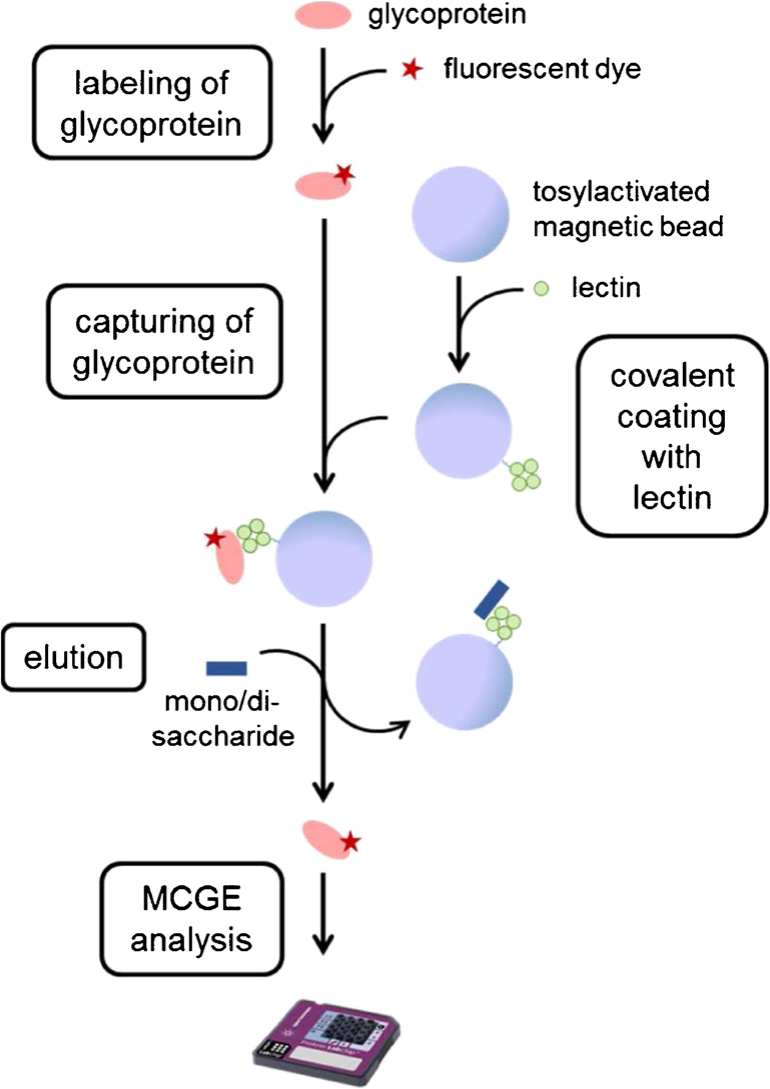
Workflow of the affinity enrichment of glycoproteins. Lectins were covalently coupled to magnetic beads and incubated with fluorescently labeled analytes. The captured glycoproteins were specifically eluted by the respective complementary mono- and disaccharides and subsequently analyzed with MCGE or SDS-PAGE

A general scheme of the specific affinity enrichment for glycoproteins combined with MCGE analysis is presented in Fig. 1. In a first step, the individual lectins ConA, WGA, and SNA were covalently coupled to tosylactivated magnetic beads. These lectin-coated beads were incubated with a sample containing glycoproteins, which had been fluorescently labeled for subsequent chip analysis. For a specific elution of the enriched glycoproteins, the respective competitive mono- or disaccharides to the lectins were applied and the eluted glycoproteins were analyzed with MCGE or, for comparison, with SDS-PAGE. A second unspecific elution step was added by incubation of the beads with denaturing buffer containing LDS or SDS to assess the degree of specific lectin and unspecific protein binding, respectively. Experiments were carried out at least in duplicates. Representative data are displayed.

### Selection of magnetic beads

**Fig. 2:**
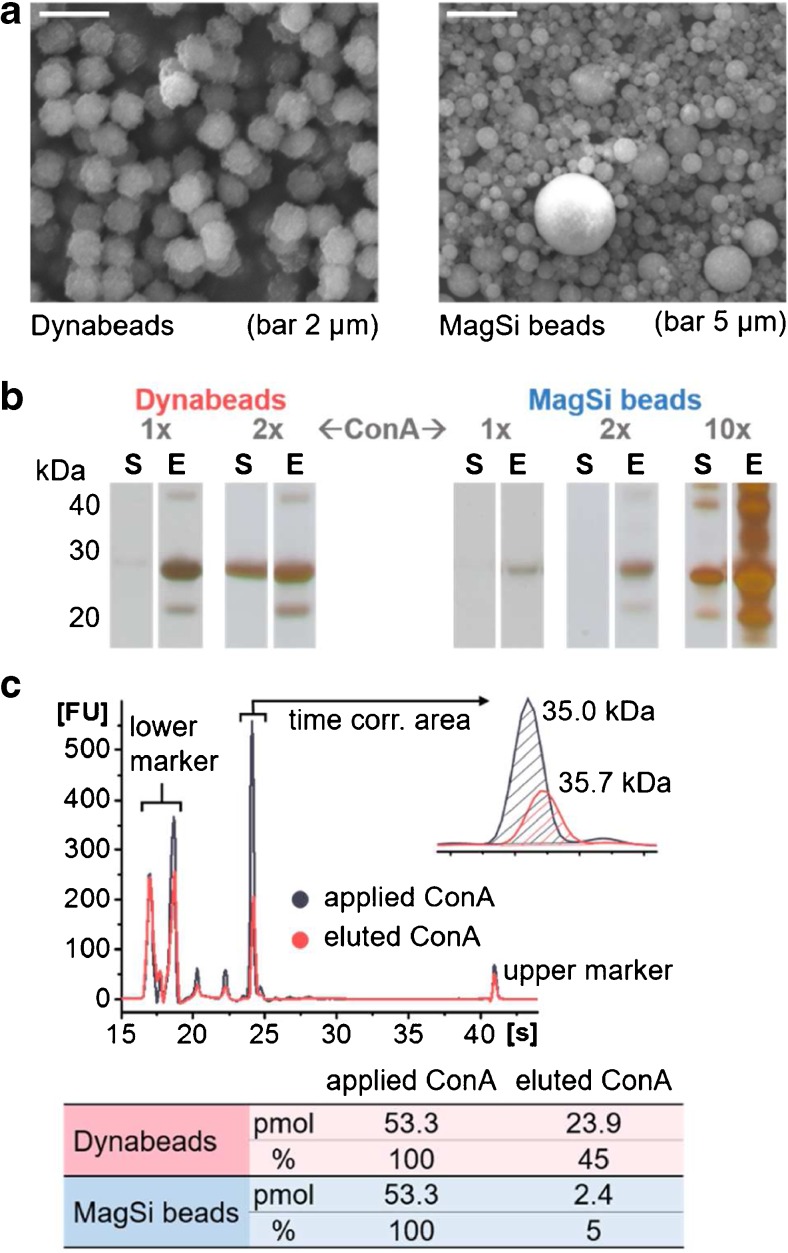
Comparison of ConA coupling reactions to tosylactivated Dynabeads and MagSi beads. (**a**) SEM analyses (FEI Quanta 200 with accelerating voltages of 10 and 20 kV, respectively) of both bead types. (**b**) SDS-PAGE analyses of the supernatant (**S**) and the unspecific elution fraction (**E**) after incubation of the beads with different amounts of ConA (1×, 5.33 μM; 2×, 10.66 μM; 10×, 53.3 μM). (**c**) Amounts of ConA (in pmol and %) in the supernatant and unspecific elution fraction as determined with MCGE (P230 assay)

Two different magnetic beads with nominal diameters of 1 μm according to manufacturers’ specifications were tested and compared. However, scanning electron microscopy experiments of the two bead materials revealed a very broad size distribution of the MagSi beads ranging from about 100 nm up to 5 μm in contrast to the rather size-uniform Dynabeads (Fig. 2a). This circumstance can influence effectiveness of enrichment and reproducibility of analyses as varying bead size distributions are directly linked to varying binding capacities.

Coupling conditions and concentrations were optimized for both beads and binding efficiencies examined using the lectin ConA. Therefore, after binding ConA to the beads, the supernatant (S) solution and the unspecific elution (E) fraction of the beads were analyzed with SDS-PAGE and MCGE (Fig. 2b, c). Remaining ConA in the supernatant was used as an indicator for a sufficient surface coverage of the bead, whereas the unspecific elution of ConA was used for assessing the amount of non-covalently bound ConA.

In the case of the Dynabeads, application of 5.33 μM ConA to 20 μg/μL beads showed a slight SDS-PAGE band in the supernatant after incubation, pointing to a sufficient lectin concentration to saturate all binding sites (Fig. 2b). Biologically active ConA is a non-covalent multimer (tetramer at physiological pH) [19]. Therefore, a protein band at the apparent molecular mass of the monomer (26 kDa) was expected to be characteristic for the unspecific elution of the beads. Figure 2b shows no band in the supernatant and only a slight band in the unspecific elution fraction of the MagSi beads when applying the same amount of lectin. This indicates that ConA monomers were almost completely linked to the bead surface without the formation of non-covalently, biologically active multimers. Similar results were observed after doubling the lectin concentration while keeping the bead concentration constant: no remaining ConA was found in the supernatant but a weak band in SDS-PAGE for the unspecific elution indicating the formation of biologically active lectin multimers. Only after application of a tenfold amount of ConA a distinct ConA band was visible in SDS-PAGE also for the supernatant indicating saturation of the magnetic bead surface.

Quantitative analysis introduced by MCGE analysis allowed the determination of ConA in the supernatant and the unspecific elution fraction after generating a calibration function for different ConA concentrations (Fig. 2c). Forty-five percent non-covalently bound ConA with regard to the initially applied amount was eluted in the case of Dynabeads and only 5% in the case of MagSi beads. From these results, it was concluded that Dynabeads need much less lectin while forming a higher number of biologically relevant multimers. Furthermore, first tests for the functionality of the immobilized lectins showed that MagSi beads exhibit a higher degree of unspecific protein binding (data not shown).

Based on these findings and with respect to the previously mentioned size heterogeneity of the MagSi beads, further optimization was restricted to the use of Dynabeads.

### Optimization of glycoprotein enrichment

For the optimization of the affinity enrichment, a set of model glycoproteins (Tf, A1AT, and AGP) with varying degrees and types of glycosylations and the non-glycosylated protein β-Gal as negative control was applied. Tf was the largest of the applied glycoproteins in terms of molecular mass but exhibited the lowest glycosylation content (one *O*-glycan, two *N*-glycans, low degree of sialylation) [20]. The smaller A1AT featured a higher glycosylation degree (one *O*-glycan, three *N*-glycans, higher number of sialylation) [21]. AGP, the smallest glycoprotein, had the highest glycan content (five *N*-glycans) and most sialic acids attached [22]. In all cases, the binding buffer contained CaCl_2_ and MnCl_2_, as these cations are required by some lectins for a successful carbohydrate recognition [19]. Next to that, the required buffer must be compatible with subsequent MCGE analyses concerning pH and concentrations of its constituents. Different time spans (15 min up to 2 h) and temperatures (4 up to 29 °C) during binding and elution steps were tested together with the addition of detergents (0.1 up to 1% Tween 20, Tween 80, Thesit, Triton X-100) to buffers, addition of extra blocking reagents (serum albumin or competitive monosaccharides) to the blocking buffer to reduce unspecific analyte binding to beads, and variations in elution reagents (acetic acid, repeated sugar elution, SDS). Least unspecific binding and elution of β-Gal were found for 1 h binding and 15 min elution steps at room temperature with no addition of detergents. Furthermore, the elution using competitive monosaccharides proved to be most specific, selectively eluting the glycoproteins but not the non-glycosylated β-Gal (see Electronic Supplementary Material (ESM) Fig. S1).

However, SDS-PAGE analysis demonstrated that glycoprotein elution was not complete. Besides glycoproteins still bound to lectin beads after specific elution also unspecifically bound β-Gal as well as non-covalently bound ConA was detected in the unspecific elution fraction. An increase of sugar concentration during specific elution did not effectively influence these results (data not shown). In favor of the higher specificity, the incomplete glycoprotein elution by using competitive sugars was accepted.

### Combination of affinity enrichment and MCGE analysis

Combining the optimized affinity enrichment with MCGE considerably reduced the required time for analysis, detection, and data evaluation (MCGE 0.5 h, SDS-PAGE > 3.5 h), as well as the applied sample amount by at least 50%. Due to enrichment buffer compatibility with MCGE buffer requirements, no additional buffer exchanges were necessary before CGE-on-a-chip analysis. Therefore, all samples could be directly analyzed after specific elution. For LIF detection, samples were fluorescently labeled with a covalent HSP-250 fluorescent dye using a minimal labeling strategy. By labeling, only the sample before the affinity enrichment, no lectin signal interfered with signals from eluted glycoprotein.

ESM Figs. S2 and S3 display the electropherograms for experiments including Tf, β-Gal, A1AT, and AGP employing ConA beads. MCGE results for samples of the initially applied glycoprotein concentrations, of the supernatant after incubation with the lectin beads, and of the specific elution fraction for the individual enrichments are shown. The direct comparison of the Tf and β-Gal enrichment (ESM Fig. S2) confirmed results from SDS-PAGE analyses. About 65% of Tf were specifically bound to the ConA beads, but also β-Gal showed a certain unspecific binding towards ConA. However, almost only Tf was eluted during the specific elution step (time corrected areas, 50:1 = Tf/β-Gal).

In addition, for A1AT and AGP, the enrichment of different sample constituents could be found. The fact that more than one signal was observed for the respective glycoproteins can be explained most likely by glycoforms but can also result from sample impurities (ESM Fig. S3). Especially in the case of A1AT, the supernatant as well as the elution fraction reveal distinct changes in the relative signal intensities. From that, a stronger binding of one constituent (77.6 kDa) to ConA could be inferred. The same results were found with SDS-PAGE analysis (ESM Fig. S1).

**Fig. 3:**
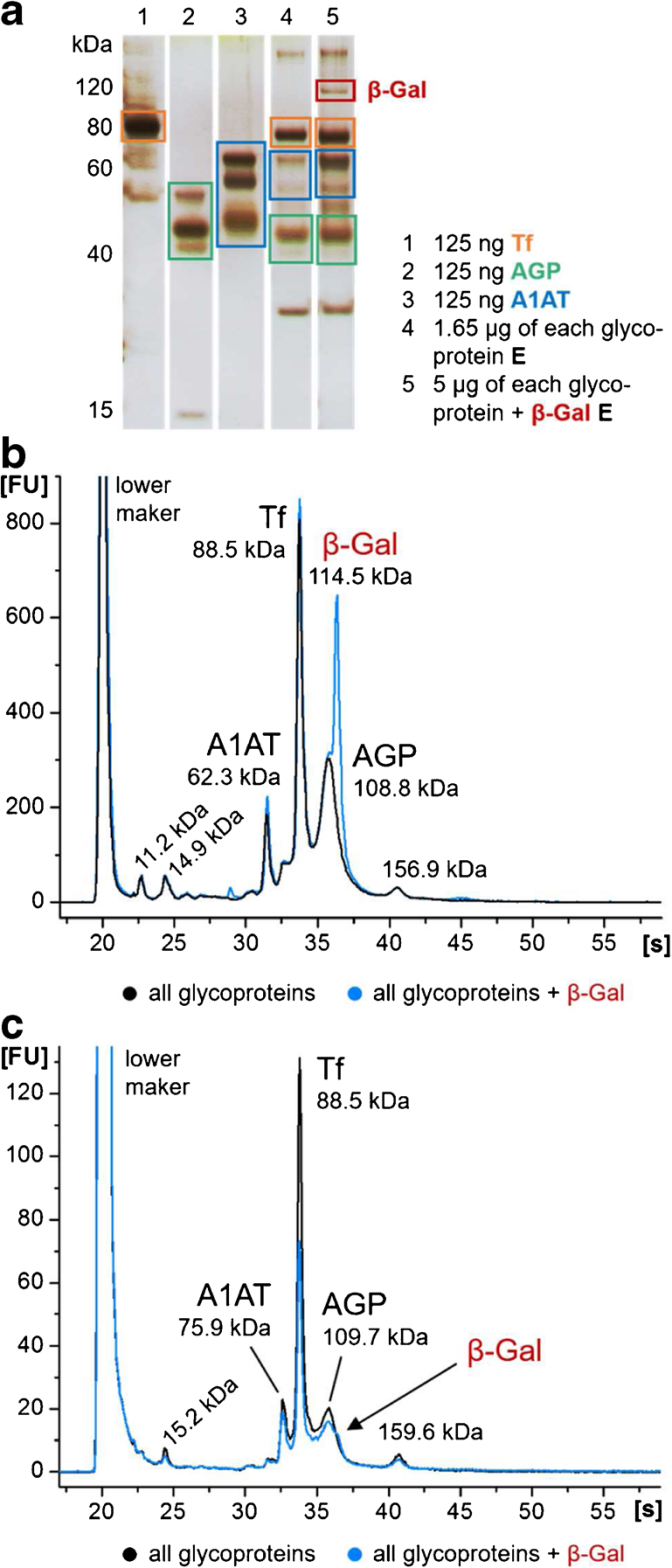
(**a**) SDS-PAGE and (**b**, **c**) MCGE (HSP-250 assay) analyses of a Con A enrichment using a mixture of the glycoproteins Tf, A1AT, and AGP with (blue line) and without (black line) β-Gal as negative control. The applied mixtures were analyzed before enrichment (**b**) and compared to the specific elution **E** (**c**)

Next to the individual affinity enrichments also, a mixture of the glycoproteins with and without the negative control was incubated with ConA beats and analyzed with MCGE and SDS-PAGE (Fig. 3). All glycoproteins could be identified before and after enrichment in the electropherograms, yet, with varying intensities depending on the presence of β-Gal. This became most obvious with Tf and AGP, which both showed lower signal intensities in the specific elution fraction, when β-Gal was added. It was found that the Tf signal was reduced by approx. 45%. For AGP, an estimation was not possible because of β-Gal co-migration; however, the reduction in signal intensity is visible. β-Gal, on the other hand, was only detectable at much lower concentration levels in the specific elution fraction, which was also confirmed by SDS-PAGE. β-Gal binding seemed to be increased in the presence of other glycoproteins compared to the individual incubation of β-Gal with ConA beads. This raised the question whether β-Gal really interacted with ConA or rather binds to other glycoproteins (“β-Gal piggyback on glycoproteins”). Such a binding also influences the binding and elution of target proteins and thus explains lower signal intensities. Nevertheless, the eluted amount of unspecifically bound negative control was small compared to the specifically bound glycoproteins as indicated by the MCGE signal intensities and SDS-PAGE band abundance, respectively.

### Selective enrichment of glycoproteins from biological samples

For the selective enrichment of glycoproteins from more complex biological samples, WGA and SNA beads were prepared together with ConA beads. All selected lectins have particular specificities to different carbohydrate moieties, and combining them allows to target a broad range of glycoforms. An equimolar mixture of the separately prepared lectin beads was used to target a high number of different glycoforms in human serum, a biological sample already well-studied in respect to glycoprotein enrichment [23]. Therefore, freshly taken blood was centrifuged, the supernatant fluorescently labeled and incubated with the mixture of lectin beads.

**Fig. 4:**
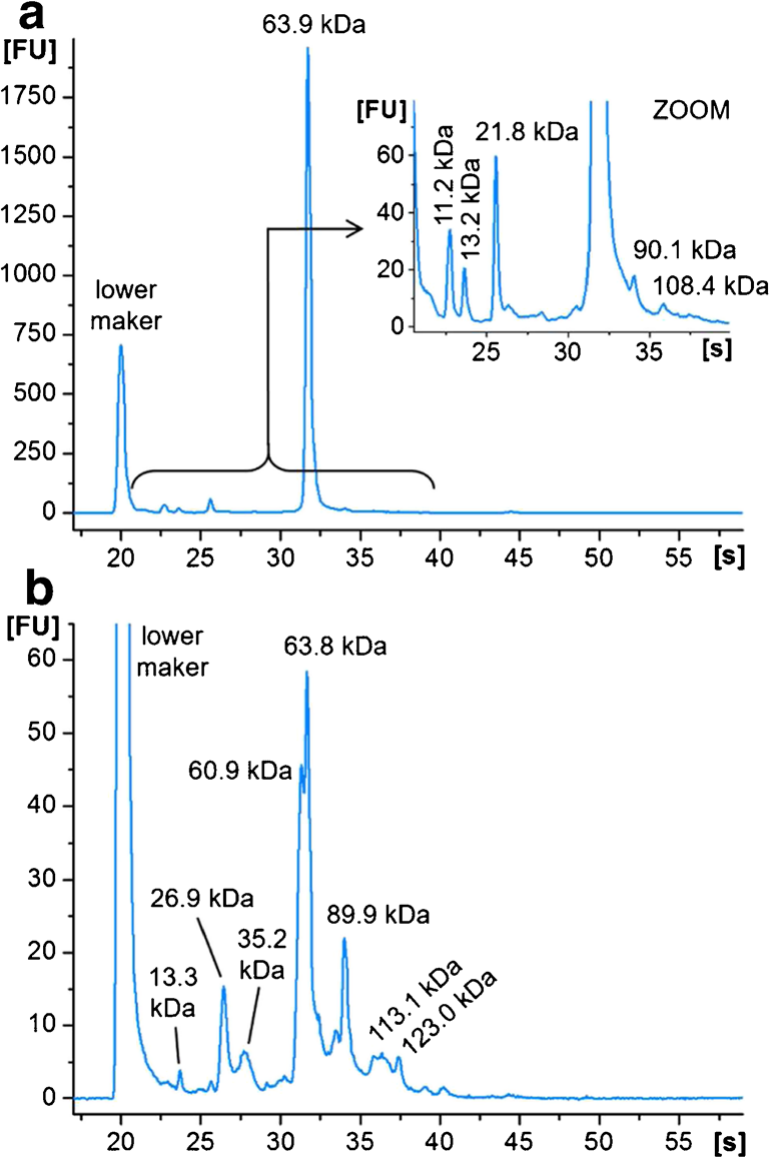
Glycoprotein enrichment of 2 μL human serum using a mixture of ConA-, SNA-, and WGA-beads (1:1:1; 2 μL each). MCGE analysis (HSP-250 assay) (**a**) before enrichment and (**b**) of the specific elution fraction **E**

Figure 4 displays the electropherograms of serum before affinity enrichment and of the specific elution fraction using a mixture of complementary sugars for each lectin. The protein profile before enrichment was dominated by a peak at 64 kDa, the most abundant protein in human serum, albumin (HSA). In contrast, the elution fraction was characterized by a completely changed peak pattern: several new components could be identified and the distinct HSA signal was strongly decreased. Unspecific interactions of HSA as a carrier protein with other components of serum, also with glycoproteins and lectins, were expected to interfere with sample enrichment, and indeed a complete depletion of the HSA peak was not achieved. However, the unspecific binding and elution of HSA could be decreased by varying the amount of applied serum and lectin beads (ESM Fig. S4).

The lowest HSA signal relative to other constituents was achieved using 8 μL of labeled serum (equivalent to 2 μL pure serum) and 6 μL of lectin beads (ConA/SNA/WGA = 1:1:1, 2 μL each) diluted in binding buffer to a final volume of 30 μL. In that ratio, 4 μL of labeled serum equals 70 μg of protein and 1 μL of lectin beads corresponds to 100 μg beads, giving a final ratio of 140 μg protein and 600 μg beads. Keeping the ratio constant but changing the concentrations of the components also influenced the peak pattern. Reducing the serum and bead concentration by 25% (4 μL labeled serum, 3 μL lectin beads, total volume 20 μL) resulted in very low overall signal intensities yet also a low HSA signal in regard to the other peaks. In contrast, increasing the concentrations by 12.5% (12 μL labeled serum, 9 μL lectin beads, total volume 40 μL) showed a relatively increased HSA peak and was thus avoided (e.g., time corrected areas, 1:2.9 = peak at 60.5 kDa:HSA, ESM Fig. S4a). Using lower concentrations for enrichment reduced sample consumption. A good compromise was reached using 2 μL of all (the three different lectin beads) components (in a total volume of 30 μL with the serum) obtaining high signal intensities and a comparably weak HSA signal (e.g., time corrected areas, 1:1.4 = peak at 60.5 kDa:HSA, Fig. 4). Changing the serum/lectin ratio by increasing the amount of beads resulted in a lower specificity of the enrichment with high HSA peaks (ESM Fig. S4b). Therefore, a mixture of 8 μL labeled serum and 6 μL lectin beads in a total volume of 30 μL was used for further experiments.

**Fig. 5:**
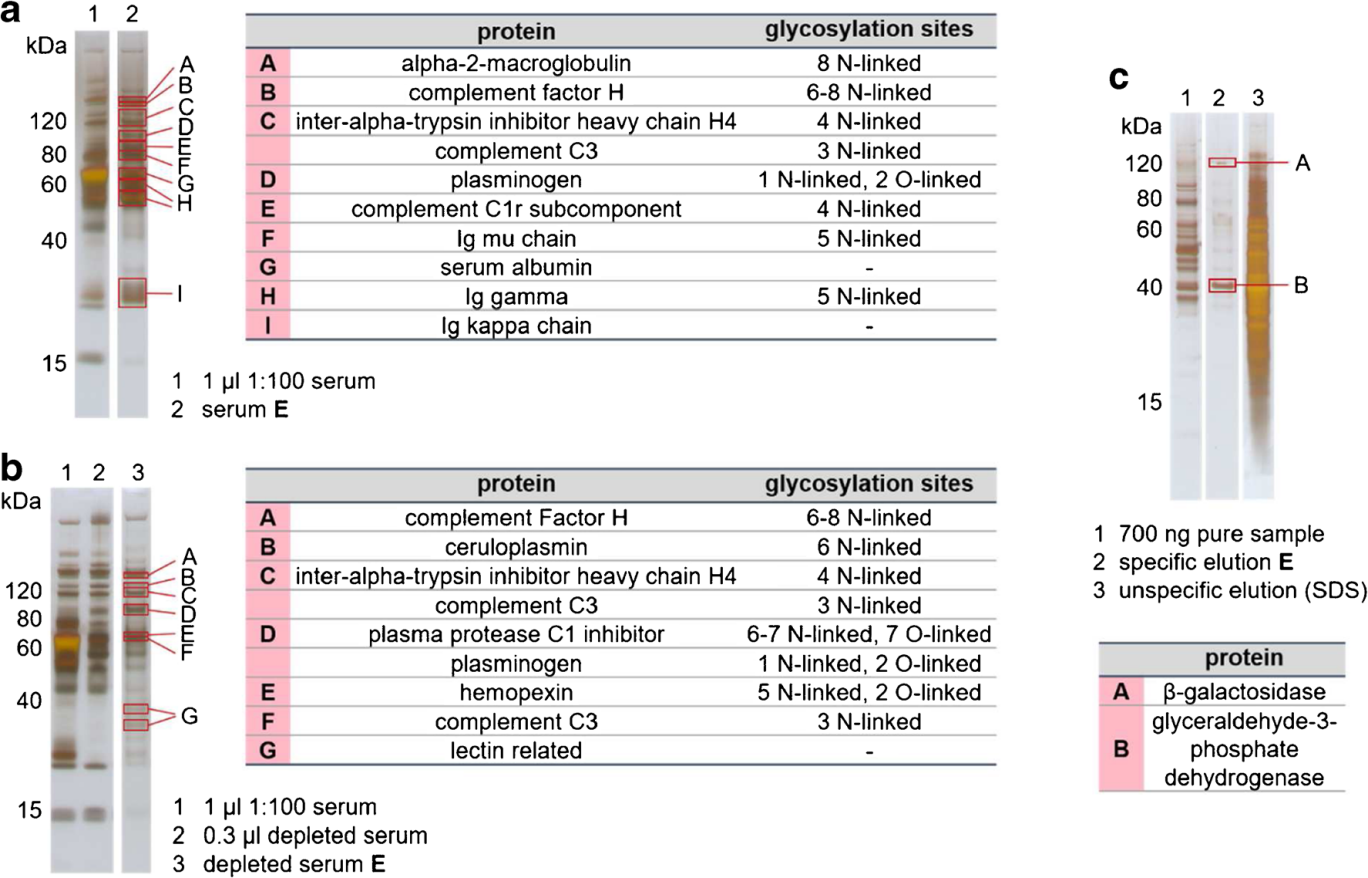
SDS-PAGE analysis of glycoproteins enriched by a lectin beads mixture (ConA/WGA/SNA 1:1:1, 2 μL each) from human serum (**a**) before and (**b**) after depletion with Pierce Top 12 spin columns and (**c**) from *T. atroviride* extract (supernatant). (**E** specific elution fraction). Proteins were identified by MALDI-MS after tryptic *in-gel* digestion

The analysis of enriched glycoproteins from serum with SDS-PAGE and subsequent tryptic *in-gel* digestion and MALDI-RTOF-MS for protein identification further demonstrated the selectivity of the method (Fig. 5a). For a better comparability, the ratios and amounts of used serum and lectin beads were kept the same as evaluated and mentioned before. Next to HSA, which was expected to be detected from previous MCGE experiments, only glycoproteins could be identified in the specific elution fractions. One has to be aware that the identified, non-glycosylated Ig kappa chain was enriched as part of the intact glycosylated antibody but separated during SDS-PAGE because of reducing conditions.

Further evaluating the selectivity of the approach, the enrichment was applied to serum depleted from the 12 most abundant serum proteins. Therefore, the serum was treated with Top 12 spin columns before incubation with the mixture of lectin beads. Figure 5b clearly shows the reduction of HSA at 69 kDa and of IgG at 56 and 27 kDa on the SDS-PAGE. Again, only glycoproteins could be identified from the depleted serum.

Furthermore, a biotechnology-oriented application regarding the cellulose degrading fungus *T. atroviride* was performed with the established strategy. The ability of filamentous fungi to secrete large amounts of glycoproteins rose the interest to use the established lectin-based enrichment approach. Although there is only little information about the nature of the glycoproteins in those fungi, the predominant forms were found to be oligomannose *N*-glycans and *O*-glycans [24]. In contrast, more complex glycan structures as present in mammalians were not detected so far. Next to that, the presence of glucose, galactose, and *N*-acetylglucosamine has been reported on the glycans. Using again a mixture of the three lectin beads, especially ConA was expected to specifically bind to the high-mannose structures of the *T. atroviride* sample. WGA should show interactions with potentially present terminal *N*-acetylglucosamines. As mainly high-mannose-type glycoproteins were detected in filamentous fungi, interactions with SNA were not particularly expected. Nevertheless, SNA was still included in the experiment in order to target a broader range of *N*-glycans.

The enrichment resulted in the identification of two proteins, glyceraldehyde-3-phosphate dehydrogenase and β-galactosidase. The latter is known to have several putative *N*-glycosylation sites in *Trichoderma reesei* [25]. Despite of the higher number of proteins being captured by the lectin beads, as shown in the unspecific elution fraction (Fig. 5c), only two were significantly eluted in the specific elution fraction. One explanation is a possible qualitative difference in the fungal glycosylation structures compared to mammalian glycoproteins and, therefore, lower binding efficiency of the lectins. On the other hand, the binding of glycoproteins to lectins is increasing with multivalency. The highly branched oligomannose structures of glycoproteins in *Trichoderma atroviride* can thus strongly bind to ConA making the elution difficult.

### Specific glycoprotein enrichment in human serum samples

**Fig. 6:**
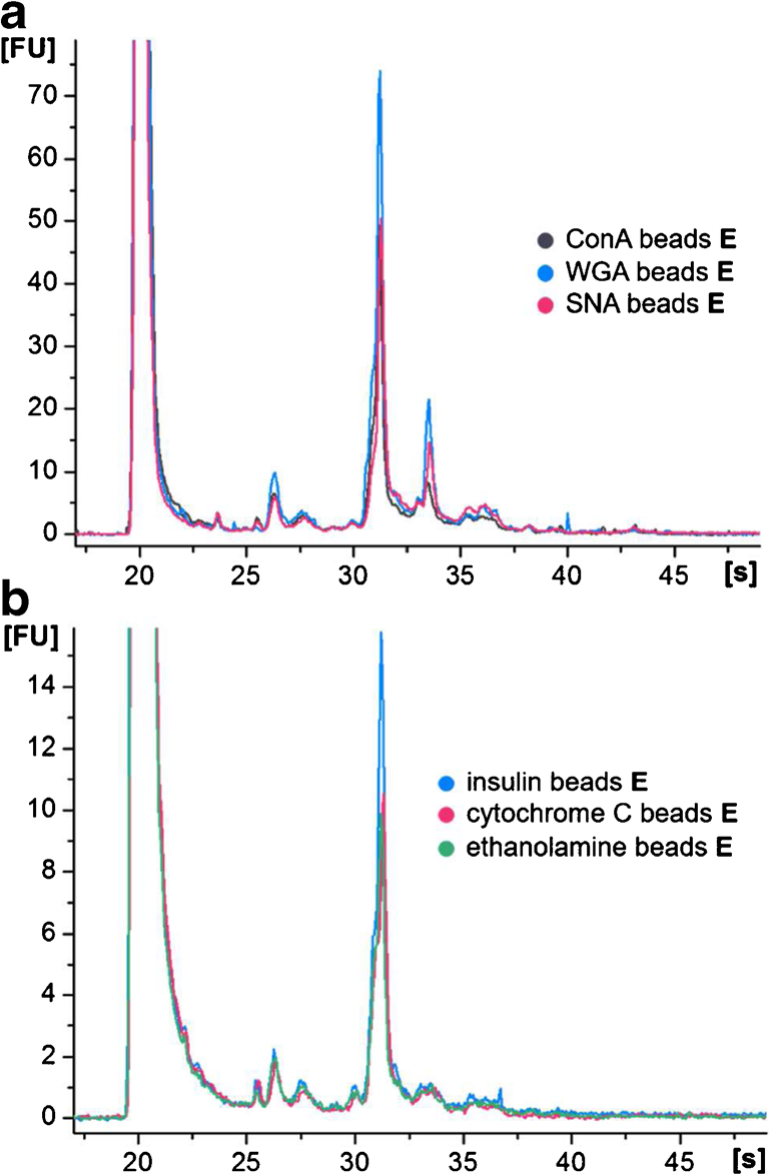
MCGE analysis (HSP-250 assay) of the specific elution fraction **E** of (**a**) human serum incubated with individual lectin beads in comparison to (**b**) beads coated with ethanolamine, insulin, or cytochrome C

As human serum showed a more complex elution profile from the lectin beads with a higher number of enriched proteins, further studies were limited to this more comprehensive sample compared to the mycelia sample. Specific glycoprotein enrichment was investigated by an individual incubation of the sample with each individual lectin bead. Against expectations, MCGE electropherograms of all three enrichment experiments showed a very similar peak pattern (Fig. 6a). Comparable results were gained in corresponding SDS-PAGE analysis (ESM Fig. S5a).

Due to this outcome, the interactions between the beads and the complex biological sample were studied in more detail. Therefore, untreated Dynabeads were incubated with human serum according to the established protocol. Resulting SDS-PAGE analysis of the specific elution fraction showed unspecific interactions of the beads with the sample (ESM Fig. S5b). Omitting covalent linking of sample constituents to the beads via free binding sites, ethanolamine was used to block all tosyl groups. By this, only the surface of the bead can interact with the sample without formation of any covalent links. Again, a similar band pattern was observed (ESM Fig. S5b). In order to reduce the surface access and thus the interaction of the sample with the bead surface, proteins of different sizes (insulin and cytochrome C) were covalently linked to the beads. However, incubation with human serum resulted again in unspecific enrichment (Fig. 6b and ESM Fig. 5b). The electropherograms of the specific elution fractions showed a similar peak pattern to the enrichment with ethanolamine blocked beads (Fig. 6b). Furthermore, the pattern was comparable to the glycoprotein enrichment using a mixture of lectin beads, however with lower intensities. Only a signal migrating at 33 s (89 kDa) was not detected.

Electrophoretic analyses suggested the enrichment of similar analytes because of comparable peak pattern when using dedicated lectin beads and others (insulin, cytochrome C, ethanolamine). However, MS identification of SDS-PAGE separated proteins gave only a few identifications for glycoproteins from the gel bands. Mostly carrier proteins, like HSA and immunoglobulins, were identified. This unspecific binding can be expected as mentioned before. On the contrary, for proteins separated by SDS-PAGE after lectin enrichment, mostly glycoproteins were identified with good significance in more than one analysis (triplicate and quadruplicate analyses of the full method, from glycoprotein enrichment to mass spectrometric identification).

It was concluded that the surface of the beads, which are made from polystyrene with a modified polyurethane layer, most likely has high potential to interact with the complex sample, i.e., the very abundant and sticky proteins. Yet, it can be considered that the unspecific interactions of proteins with the beads might be reduced due to shielding effects of the bound lectins of the surface, but it was still recognizable (HSA binding). As a consequence, this information necessarily has to be taken into account for any data interpretation in regard to specificity investigations.

## Conclusions

The present study shows the successful combination of lectin affinity enrichment with MCGE for a sensitive and rapid glycoprotein analysis. Results were in very good agreement with corresponding SDS-PAGE findings, but exhibiting as major advantages lower sample consumption and taking much shorter time to the final result distinguishing our described setup for method development. Additional MS identification proved the selectivity of the method even for complex biological samples. However, the existence of unspecific interactions between analytes (in very complex matrices as serum) and the magnetic beads (particular the evaluated Dynabeads) were observed, which makes certain analyses challenging. A more detailed study by, e.g., 2-D gel electrophoresis (on the intact glycoprotein level) or HPLC-ESI-MS (on the enzymatic generated peptide/glycopeptide level) will be necessary for in-depth structural studies of lectin selectivities and specificities with magnetic beads. The combination of the bead-based lectin affinity enrichment with MCGE proved to be a strategy opening up new ways in 2-D gel electrophoresis or particular QC and PAT in biotechnology. Furthermore, it forms the base for the future development of a true lab-on-a-chip system, i.e., lectin enrichment on the chip with multiple lectin selectivities combined with capillary electrophoresis and on-line ESI-MS (similar to the recently introduced ZipChip 908 device [26]).

## Electronic supplementary material

ESM 1 (PDF 800 kb)

## References

[1] Apweiler R, Hermjakob H, Sharon N (1999). On the frequency of protein glycosylation, as deduced from analysis of the SWISS-PROT database. Biochim Biophys Acta.

[2] Varki A (1993). Biological roles of oligosaccharides: all of the theories are correct. Glycobiology.

[3] Dan X, Liu W, Ng TB (2016). Development and applications of lectins as biological tools in biomedical research. Med Res Rev.

[4] Syed P, Gidwani K, Kekki H, Leivo J, Pettersson K, Lamminmaki U (2016). Role of lectin microarrays in cancer diagnosis. Proteomics.

[5] Mechref Y, Madera M, Novotny MV (2008). Glycoprotein enrichment through lectin affinity techniques. Methods Mol Biol.

[6] Fanayan S, Hincapie M, Hancock WS (2012). Using lectins to harvest the plasma/serum glycoproteome. Electrophoresis.

[7] Mandal DK, Bhattacharyya L, Koenig SH, Brown RD, Oscarson S, Brewer CF (1994). Studies of the binding specificity of concanavalin A. Nature of the extended binding site for asparagine-linked carbohydrates. Biochemistry.

[8] Schwefel D, Maierhofer C, Beck JG, Seeberger S, Diederichs K, Moller HM (2010). Structural basis of multivalent binding to wheat germ agglutinin. J Am Chem Soc.

[9] Shahidi-Noghabi S, Van Damme EJ, Smagghe G (2008). Carbohydrate-binding activity of the type-2 ribosome-inactivating protein SNA-I from elderberry (*Sambucus nigra*) is a determining factor for its insecticidal activity. Phytochemistry.

[10] Loo D, Jones A, Hill MM (2010). Lectin magnetic bead array for biomarker discovery. J Proteome Res.

[11] Yang Z, Hancock WS (2004). Approach to the comprehensive analysis of glycoproteins isolated from human serum using a multi-lectin affinity column. J Chromatogr A.

[12] Bousse L, Mouradian S, Minalla A, Yee H, Williams K, Dubrow R (2001). Protein sizing on a microchip. Anal Chem.

[13] Kuschel M, Neumann T, Barthmaier P, Kratzmeier M (2002). Use of lab-on-a-chip technology for protein sizing and quantitation. J Biomol Tech.

[14] Wenz C, Marchetti-Deschmann M, Herwig E, Schrottner E, Allmaier G, Trojer L (2010). A fluorescent derivatization method of proteins for the detection of low-level impurities by microchip capillary gel electrophoresis. Electrophoresis.

[15] Engel N, Weiss VU, Wenz C, Rufer A, Kratzmeier M, Glück S (2015). Challenges of glycoprotein analysis by microchip capillary gel electrophoresis. Electrophoresis.

[16] Shevchenko A, Wilm M, Vorm O, Mann M (1996). Mass spectrometric sequencing of proteins silver-stained polyacrylamide gels. Anal Chem.

[17] Blum H, Beier H, Gross HJ (1987). Improved silver staining of plant proteins, RNA and DNA in polyacrylamide gels. Electrophoresis.

[18] Perkins DN, Pappin DJ, Creasy DM, Cottrell JS (1999). Probability-based protein identification by searching sequence databases using mass spectrometry data. Electrophoresis.

[19] Mandal DK, Kishore N, Brewer CF (1994). Thermodynamics of lectin-carbohydrate interactions. Titration microcalorimetry measurements of the binding of N-linked carbohydrates and ovalbumin to concanavalin A. Biochemistry.

[20] Giménez E, Benavente F, Barbosa J, Sanz-Nebot V (2007). Towards a reliable molecular mass determination of intact glycoproteins by matrix-assisted laser desorption/ionization time-of-flight mass spectrometry. Rapid Commun Mass Spectrom.

[21] Sturiale L, Barone R, Palmigiano A, Ndosimao CN, Briones P, Adamowicz M (2008). Multiplexed glycoproteomic analysis of glycosylation disorders by sequential yolk immunoglobulins immunoseparation and MALDI-TOF MS. Proteomics.

[22] Nakano M, Kakehi K, Tsai MH, Lee YC (2004). Detailed structural features of glycan chains derived from alpha1-acid glycoproteins of several different animals: the presence of hypersialylated, O-acetylated sialic acids but not disialyl residues. Glycobiology.

[23] Madera M, Mechref Y, Klouckova I, Novotny MV (2006). Semiautomated high-sensitivity profiling of human blood serum glycoproteins through lectin preconcentration and multidimensional chromatography/tandem mass spectrometry. J Proteome Res.

[24] Conesa A, Punt PJ, van Luijk N, van den Hondel CAMJJ (2001). The secretion pathway in filamentous fungi: a biotechnological view. Fungal Genet Biol.

[25] Gamauf C, Marchetti M, Kallio J, Puranen T, Vehmaanpera J, Allmaier G (2007). Characterization of the bga1-encoded glycoside hydrolase family 35 beta-galactosidase of *Hypocrea jecorina* with galacto-beta-D-galactanase activity. FEBS J.

[26] Khatri K, Klein JA, Haserick JR, Leon DR, Costello CE, McComb ME, Zaia J (2017). Microfluidic capillary electrophoresis-mass spectrometry for analysis of monosaccharides, oligosaccharides, and glycopeptides. Anal Chem.

