# Supplementary material for: Microchip capillary gel electrophoresis combined with lectin affinity enrichment employing magnetic beads for glycoprotein analysis
Source: Anal Bioanal Chem. 2017 Sep 20;409(28):6625–34. doi: 10.1007/s00216-017-0615-0 (PMC5670189; doi:10.1007/s00216-017-0615-0)
Supplement: Supplementary file 1 — (PDF 800 kb) [file 216_2017_615_MOESM1_ESM.pdf]

Analytical and Bioanalytical Chemistry

Electronic Supplementary Material

Microchip capillary gel electrophoresis combined with lectin affinity enrichment employing magnetic beads for glycoprotein analysis

Nicole Y. Engel, Victor U. Weiss, Christian Wenz, Susanne Glück, Andreas Rüfer, Martin Kratzmeier, Martina Marchetti-Deschmann, Günter Allmaier

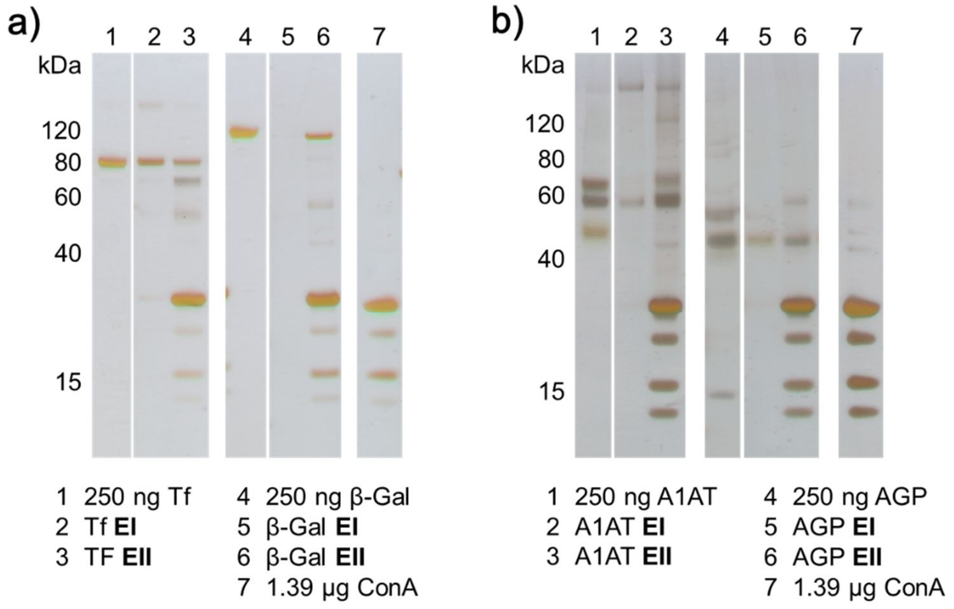

Fig. S1 SDS-PAGE analysis of a ConA enrichment of the glycoproteins (a) Tf (lane 1-3), (b) A1AT (lane 1-3), and (b) AGP (lane 4-6), as well as of (a) the non-glycosylated β-Gal (lane 4-6) using ConA-beads. The samples were analysed before (250 ng protein each) and after enrichment (specific elution **EI**, unspecific elution **EII**)

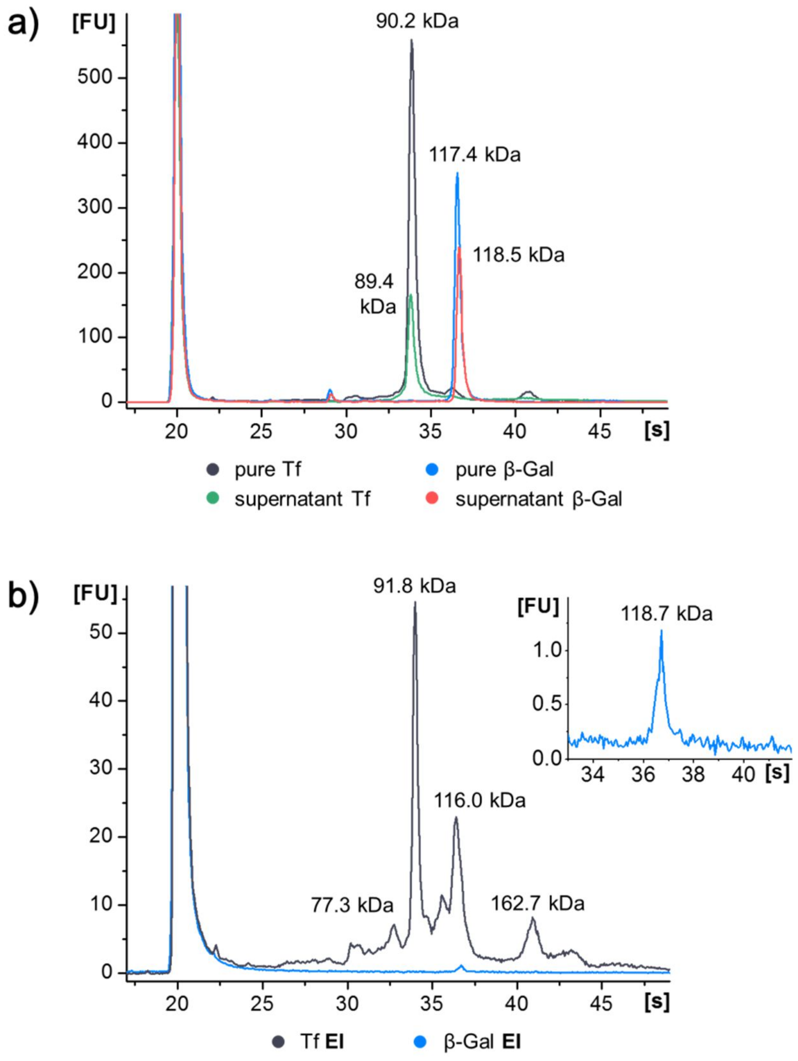

Fig. S2 MCGE (HSP-250 assay) analyses of the ConA enrichments of the glycoprotein Tf and the non-glycosylated β-Gal. The samples were analysed before (a: initially applied protein) and after enrichment (a: supernatant, b: specific elution fraction **EI**)

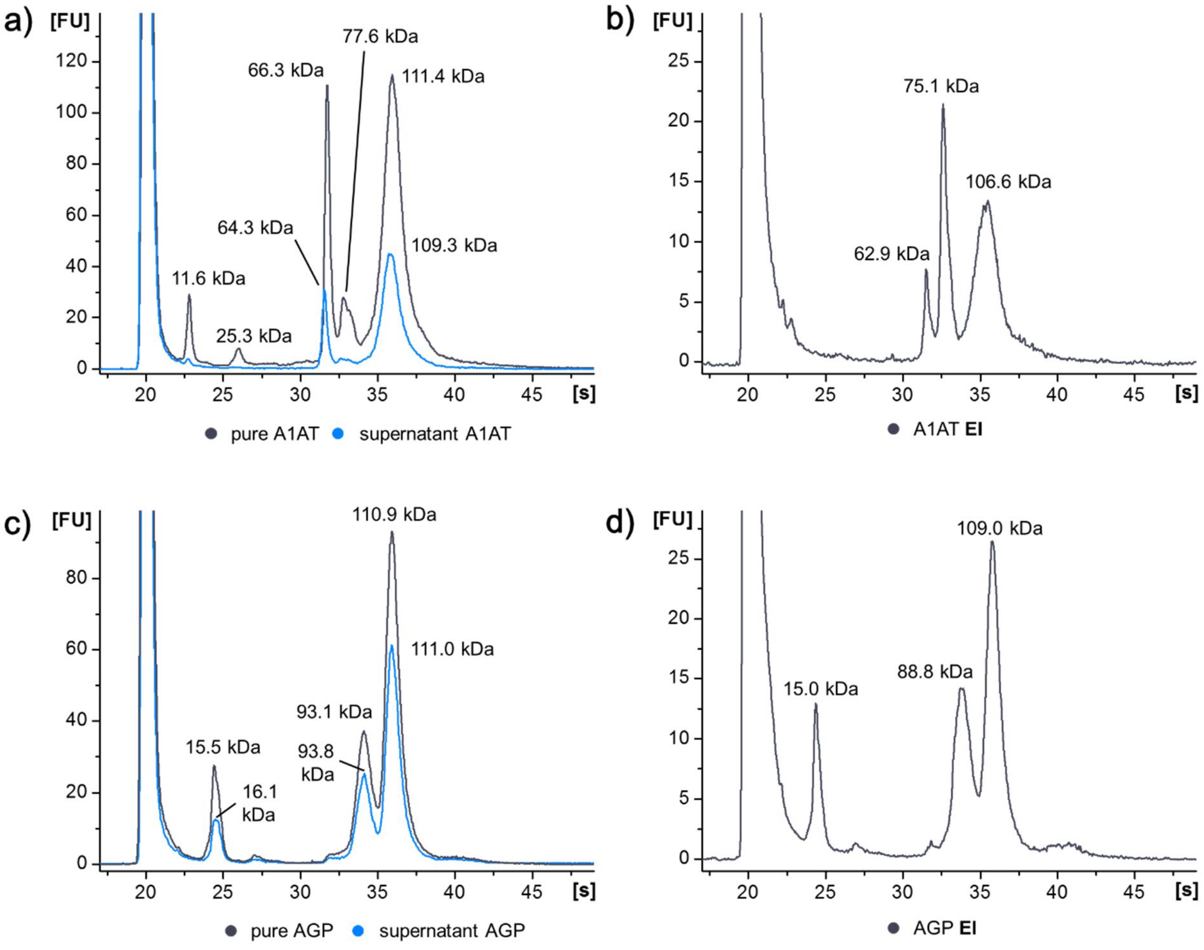

Fig. S3 MCGE (HSP-250) analyses of ConA enrichments of the glycoproteins (a/b) A1AT and (c/d) AGP. The samples were analysed before (a/c: initially applied protein) and after enrichment (a/c: supernatant, b/d: specific elution fraction)

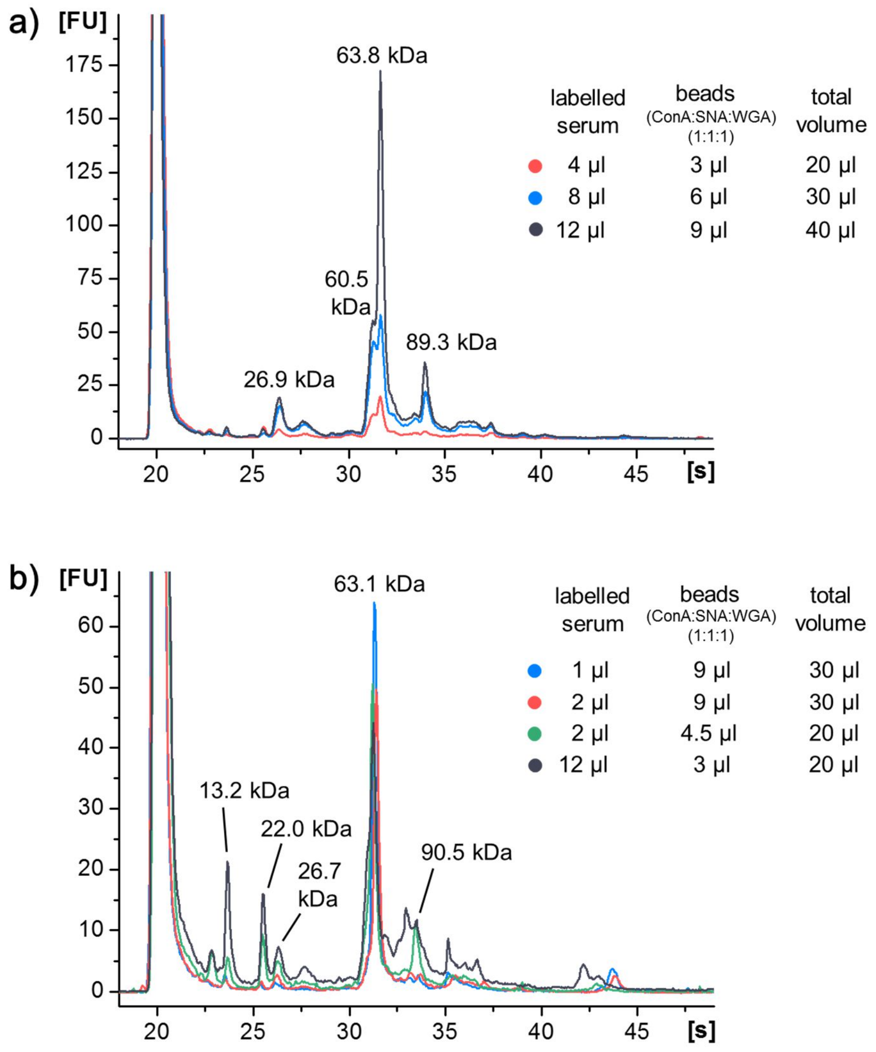

Fig. S4 Enrichment of human serum with a mixture of lectin beads with (a) varying serum/bead concentrations or (b) serum/bead ratios. Specific elution fractions were analysed by MCGE using the HSP-250 assay

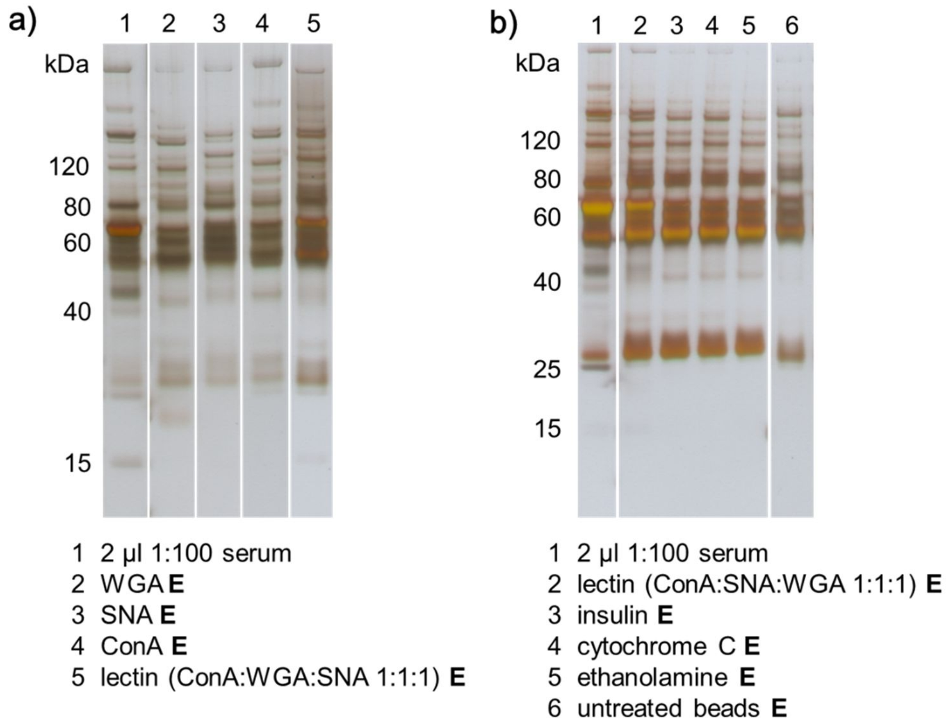

Fig. S5 Human serum was incubated (a) with each lectin bead individually and (b) with beads coated with several analytes of different sizes and properties. The specific elution fractions **E** of each enrichment were analysed by SDS-PAGE
